# The effect of neurologic music therapy in patients with cerebral palsy: A systematic narrative review

**DOI:** 10.3389/fneur.2022.852277

**Published:** 2022-09-13

**Authors:** Seoyon Yang, Jee Hyun Suh, SuYeon Kwon, Min Cheol Chang

**Affiliations:** ^1^Department of Rehabilitation Medicine, College of Medicine, Ewha Woman's University, Seoul, South Korea; ^2^Department of Physical Medicine and Rehabilitation, College of Medicine, Yeungnam University, Daegu, South Korea

**Keywords:** music, music therapy, cerebral palsy, rehabilitation, motor, neurologic music therapy

## Abstract

**Background:**

Cerebral palsy (CP) is one of the most common causes of disability in children. It is characterized by impairment in motor function and coordination and difficulties in performing daily life activities. Previous research supports that neurologic music therapy (NMT) was effective in improving motor function, cognition, and emotional wellbeing in patients with various neurologic disorders. However, the benefit of NMT in patients with CP have not yet been thoroughly investigated. The aim of this review was to investigate the potential effect of NMT motor rehabilitation techniques for patients.

**Materials and methods:**

We searched articles published up to May 24, 2022 in PubMed, Embase, Scopus, Cochrane library, Web of science, and Ovid MEDLINEdatabases. We included studies that investigated the effect of NMT in patients with CP.

**Results:**

After search, 4,117 articles were identified using the search terms. After reading the titles and abstracts, 4,089 articles that did not meet our inclusion criteria were excluded. The remaining 28 articles which were assessed for eligibility. Finally, 15 studies were included in this systematic review. Among 15 studies that investigated the effect of NMT on patients with CP, 7 studies were on rhythmic auditory stimulation (RAS), 6 studies were on therapeutic instrumental music performance (TIMP), and 2 studies were on patterned sensory enhancement (PSE).

**Conclusions:**

Various techniques of NMT brings beneficial effects for gross and fine motor improvements in patients with CP. NMT techniques, such as RAS, TIMP, and PSE, may be a potential alternative rehabilitation strategy to enhance gross and fine motor skills for patients with CP.

## Introduction

Cerebral palsy (CP) is a non-progressive motor disorder caused by injury to brain structures or abnormal development of the brain (1). It is one of the most common causes of motor disability in children (2). Depending on the location of the brain injury and degree of severity, disabilities may involve domains of motor, sensory, cognition, communication, and behavior (3). The main features of CP are problems in movement, posture, balance, upper body coordination and functions (4). It may also show sensory impairments, attentional deficits, learning difficulties, deficits in language development, cognition, perception, epilepsy, impaired manual skills, and orthopedic problems, such as spasticity and weakness (5, 6). Impairment of motor skills may limit independence in activities of daily living and everyday functioning in patients with CP. Therefore, effective rehabilitative training programs targeting improvement of motor abilities and goal-directed function are often necessary. Conventional rehabilitative programs focus on normal motor development, and additional therapeutic approaches that involve multiple stimuli and processes, such as music based interventions, may help to enhance motor learning and sensory-motor integration in patients with CP (7).

Neurologic music therapy (NMT) is defined as the therapeutic approach for motor, sensory, cognitive, and language dysfunctions due to disease or injury to the human nervous system based on neuroscientific models of music perception and music production (8). It uses music as a rehabilitative cue to induce various brain responses (9). There are several techniques used in NMT, such as Rhythmic Auditory Stimulation (RAS), therapeutic instrumental music performance (TIMP), and Patterned Sensory Enhancement (PSE), which target specifically motor function (8). NMT uses music as a versatile stimulus for the brain, and is suggested as a promising complementary strategy when applied in combination with other therapeutic programs in neurologic disorders (10). Music is used as a therapeutic tool to promote multimodal activation of the brain and to induce neuroplastic changes in dysfunctional and impaired networks of the damaged brain (11). It may increase neuroplasticity by consistent bidirectional transmission of information between sensory and motor areas of brain through music (12). Music activities may improve motor planning, coordination and communication skills, and it may also facilitate learning, and provide an emotional experience (9). NMT can bring positive effects during rehabilitation by providing auditory and visual feedback and connecting motor and cognitive aspects (13, 14).

Previous research supports that NMT was effective in improving motor function, cognition, and emotional wellbeing in patients with stroke, dementia, Parkinson's disease, epilepsy, or multiple sclerosis (15–19). However, the overall benefit of NMT in patients with CP have not yet been thoroughly investigated. Patients with CP may suffer from permanent disabilities and continuous rehabilitation programs may be necessary to these patients. The aim of this review was to summarize the effect of NMT for patients with CP, and to suggest another possible therapeutic option besides conventional rehabilitation method to improve gross and fine motor functions for patients with CP.

## Methods

A systematic review of studies which investigated the effect of NMT in patients with CP was conducted.

### Search strategy

This systematic review was performed according to the Preferred Reporting Items for Systematic Reviews and Meta- Analyses (PRISMA) guidelines (20). We systematically searched for relevant articles published up until May 24, 2022, using PubMed, Embase, Scopus, Cochrane library, Web of science, and Ovid MEDLINE databases. The following keywords were used in the search “music^*^,” “music therapy,” “neurologic music therapy,” “cerebral palsy,” “cerebral pals^*^,” and “CP.”

### Study selection

The eligibility criteria for this review was informed by the Population Intervention Comparator Outcome (PICO) framework (21). Studies which investigated the effect of NMT on patients with CP were included. The main outcome of interests were the improvement of gross motor, fine motor, and function. We applied the following inclusion criteria for the selection of articles: (1) patients with CP; (2) the effect of NMT was investigated; (3) outcomes as the improvement of gross motor, fine motor, and function. The exclusion criteria were as follows: (1) studies were not related to patients with CP or NMT (2) reviews, case reports, commentaries, letters, and animal studies; (3) study outcomes were not reported, insufficient or not related to motor symptoms of CP patients; (4) studies not published in English. Two independent reviewers excluded articles that were irrelevant after reading titles and abstracts (SYY and MCC) and full-text assessments were done for final inclusion. The reviewers attempted to resolve any disagreement by consensus. The opinion of a third reviewer (JHS) was put into consideration to resolve the disagreement if necessary.

### Data extraction

All data were extracted independently by two reviewers (SYY and MCC) using a standard data collection form. The following data were recorded using a table from each eligible article: (1) the name of the first author, (2) year of publication, (3) number of patients, (4) type of NMT intervention, (5) results.

### Quality assessment

Two reviewers (SYY and MCC) independently assessed the risk of bias according to the criteria of the Cochrane Handbook for Systematic Reviews of Interventions for the included randomized controlled trials (22) and the Risk Of Bias In Non-randomized Studies-of Interventions (ROBINS-I) tool for the included non-randomized trials (23). ROBINS-I evaluated the following specific domains: bias from confounding, bias from the process of participant selection, bias due to classification of interventions, bias due to deviations from intended interventions, bias from missing data, bias from measurement of outcomes, and bias from selection of the reported results. Judgments of bias were expressed as “low risk,” “high risk,” or “unclear risk.” Two reviewers (SYY and MCC) independently assessed the risk of bias in each domain and the reviewers discussed discrepancies until a consensus was reached.

## Results

**Figure 1:**
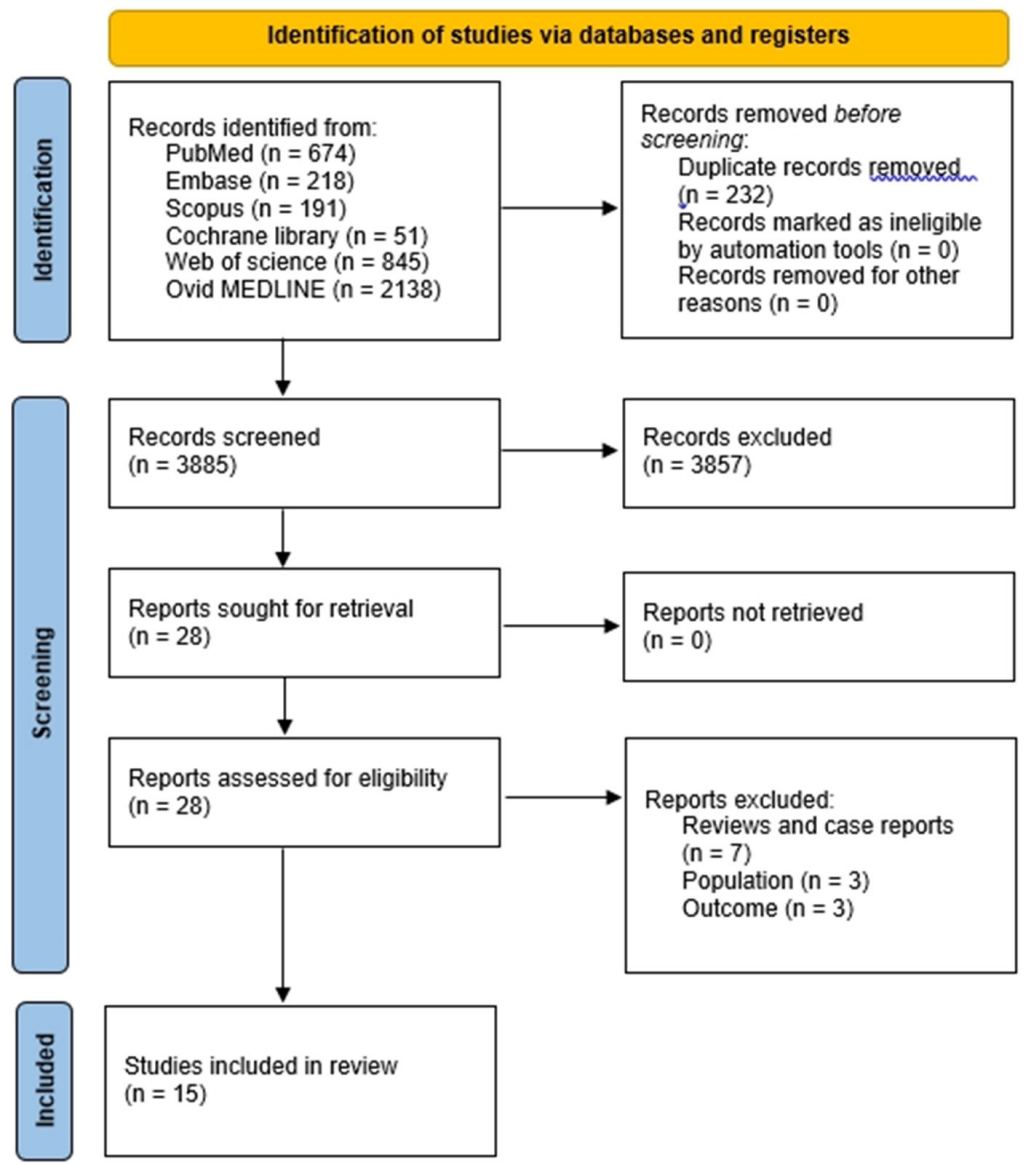
Flow diagram of the study selection process.

After search, 4,117 articles were identified. After reading the titles and abstracts, we excluded 4,089 out of 4,117 articles that did not meet our inclusion criteria (Figure 1). The remaining 28 articles were assessed for eligibility, and 13 articles were excluded due to following reasons: 7 studies were review literatures and case reports, 3 studies did not involve patients with CP, 3 studies did not report the outcome with sufficient data. Finally, 15 articles that investigated the effect of NMT on patients with CP were included in this systematic review. We then further analyzed the type of NMT intervention, number of patients, detailed method of the NMT, and the effect of NMT.

### Study characteristics

We categorized 15 studies according to the type of NMT interventions: RAS, TIMP, and PSE. RAS is a training method that uses external auditory cues to guide movement through anticipated temporal sequence to enhance motor skills by providing rhythmic stimulation to the motor center of the brain (24). TIMP uses musical instruments to exercise and facilitate functional movement patterns (25). PSE uses various musical elements, such as rhythmic, melodic, harmonic, and dynamic-acoustical elements, as cues for movement patterns (26). Among these 15 studies, 7 studies were on RAS (27–33), 6 studies were on TIMP (7, 12, 25, 34–36), and 2 studies were on PSE (37, 38). Ten studies were conducted on children and adolescents with CP (7, 12, 25, 27, 28, 32, 34, 36–38) and five studies were conducted on adults with CP (29–31, 33, 35). The characteristics of the studies, including total number of patients, age, type of CP (if mentioned), type of NMT evaluation tools, treatment duration, follow-up period, and results, are summarized in Table 1.

**Table 1:** Characteristics of included studies.

**No**.	**References**	**Study design**	**Number of patients**	**Age**	**Type of music therapy**	**Evaluation**	**Treatment duration**	**Follow-up period**	**Results**
1	Dogruoz Karatekin and Icagasioglu (25)	Non-RCT	9 adolescent CP vs. 9 healthy controls	12.33 ± 1.58 vs. 12.44 ± 1.67	TIMP (piano training)	Upper limb function (MACS, Box Block Test, Nine-Hole Peg Test, dynamometer and key pressing force of fingers)	40 min, 2 times/week for 3 months	Before and 3 months after	After piano training, significant improvements were observed in MACS box block test, nine-hole peg test, and key pressing powers of all fingers, especially 4th and 5th fingers.
2	Kim et al. (33)	Non-RCT	13 adult diplegic CP (6 simple vs. 7 complex chords)	20.0 ± 2.8 vs. 19.5 ± 5.0	RAS (simple vs. complex chords)	Gait analysis	30 min, 3 times/week for 4 weeks (12 sessions)	2 days before and 2 days after	After RAS, cadence, velocity, and stride length significantly increased, but no significant group effect was found. The complex RAS group showed increased maximal ankle plantar flexion in the preswing phase.
3	Marrades-Caballero et al. (12)	RCT	18 children severe bilateral CP vs. 9 control	4–18 years	TIMP (+phyisotherapy vs. physiotherapy)	Upper limb function (Chailey skill levels), gross motor	40 min, 1 time/week, 16 weeks (13 sessions)	4 months	Significant improvements in the overall and specific “arm and hand position” and “activities” from the Chailey Levels of Ability and the locomotor stages were observed in the group which received the music therapy.
4	Ben-Pazi et al. (32)	RCT	18 children with CP (9 intervention vs. 9 control)	7.5 ± 44.1	Auditory stimulation with music vs. music alone	Fine and gross motor function	10–30 min, 4 times/week for 4 weeks	5 months	Children receiving auditory stimulation attained more goals than children who listened to music alone. Upper extremity skills improved in the study group. Similar gross motor function changes were documented in both groups.
5	Alves-Pinto et al. (7)	Non-RCT	9 adolescent with CP vs. 7 adults with CP vs. 6 healthy adolescents	Adolescent: mean 15.0, adults: mean 44.0	TIMP (short-term piano training)	Hand motor function, perception of vibration	1 h, 2 times/week for 4 weeks (total of 8 h)		A significant effect of training on the ability to perceive the localization of vibrations over fingers was found. No significant changes in the regularity of keystrokes.
6	Efraimidou and Proios (31)	RCT	10 adults with CP (5 RAS vs. 5 control)	35.2 ± 13.0 vs. 38.8 ± 12.3	RAS	Gait (Timed Up and Go test, 10 m walk test), balance (Berg balance scale), psychological parameters (Self Esteem Scale questionnaire, Profile of Mood States)	50 min, 2 times/week, 8 weeks (16 sessions)		The results showed differences on gait, balance and psychological parameters were statistically significant after music program.
7	Lampe et al. (36)	Non-RCT	10 children with CP	6–16 yrs	TIMP (piano training)	Hand motor function (piano test, Box-and-Block test, hand dynamometer test)	35–40 min, 2 times/week for 18 months		The analysis showed a significant improvement in the uniformity of keystrokes during the training. No significant changes were detected by the Box-and-Block test and grip strength test.
8	Chong et al. (35)	Non-RCT	5 adults with CP	20–39 yrs	TIMP	Hand motor function	30 min, 2 times/week for 6–9 weeks (12 sessions)	Before and after	The velocity of key pressing force increased to a significant level, and the second and fifth fingers improved to a greater degree after continuous keyboard training.
9	Wang et al. (38)	RCT	36 children with CP (18 PSE vs. 18 control)	9.00 ± 1.99 vs. 8.98 ± 2.61	PSE	GMFM, PEDI mobility and self-care domains, 1 RM of sit-to-stand, walking speed	6 weeks	Before, after, 6, 12 weeks	The PSE group improved significantly greater than control group in the GMFM D and Goal dimensions after training and the improvement persisted for at least 6 or 12 weeks.
10	Kim et al. (30)	RCT	28 adults with CP with bilateral spasticity (15 RAS vs. 13 NDT)	27.3 ± 2.4 vs. 27.3 ± 2.5	RAS	Gait analysis	3 times/week for 3 weeks	Before and after	RAS significantly increased cadence, walking velocity, stride length, and step length. Anterior tilt of the pelvis and hip flexion during a gait cycle was significantly ameliorated after RAS. Gait deviation index also showed modest improvement in CP patients treated with RAS.
11	Hamed and Abd-elwahab (28)	RCT	30 children with CP (15 music vs. 15 control)	7.03 ± 0.76 vs. 7.07 ± 0.82	Pedometer with music	Gait analysis	3 times/week for 3 mo	Before and after 3 mo	Gait parameters, including velocity, stride length, and cadence, showed statistically significant improvement in patients who received pedometer-based gait training.
12	Kim et al. (29)	Non-RCT	14 adult CP with bilateral spasticity vs. 30 healthy controls	25.64 ± 7.31 vs. 21.50 ± 1.74	RAS (with vs. without RAS)	Gait analysis	NA	Before and after	RAS resulted in kinematic changes of the pelvic and hip movement in spastic CP. RAS was associated with improvement in gait pathology and temporospatial asymmetry in household ambulators.
13	Peng et al. (37)	RCT	23 children with CP	8.7 ± 2.0	PSE (with PSE first 5 repetitions vs. without 3 repetitions vs. no music)	Gross motor function (loaded sit-to-stand)	NA	Before and after	In the PSE condition, improvement in peak knee extensor power, total extensor power, and center-of-mass smoothness were observed, which showed that individualized PSE music helped to improve the performance of STS.
14	Nasruddin (34)	Non-RCT	9 children with CP	7–12 yrs	TIMP (Gamelan music-percussive instruments)	Attention span and concentration and gross motor function	1 h, 2 times a week	Before and after	All subjects showed memory improvement. At post-test, all subjects scored significantly higher on gross motor function as measured by standing. They also showed significant improvement over time on the measures of walking, jumping, and running.
15	Kwak (27)	Non-RCT	25 children with spastic CP (7 self-guided, 9 therapist-guided, 9 control group)	6–20 yrs	RAS	Gait analysis	30 min, 5 times a week for 3 weeks	Before and after	Therapist-guided training group with RAS showed a statistically significant difference in stride length, velocity, and asymmetry.

CP, cerebral palsy; GMFM, Gross Motor Function Measure; NA, not applicable; NDT, neurodevelopmental treatment; nRCT, non-RCT; PSE, patterned sensory enhancement; RAS, rhythmic auditory stimulation; RCT, randomized controlled trials; TIMP, therapeutic instrumental music performance; STS, sit-to-stand exercise.

Some studies compared the effect of NMT with controls (7, 12, 25, 27–29, 31, 32, 38), but other studies were observational studies without a control group for comparison (34–37). One study compared two methods of RAS using simplex *versus* complex chords (33) and another study compared RAS with traditional method of rehabilitation (NMT) (30).

### Risk of bias

**Figure 2:**
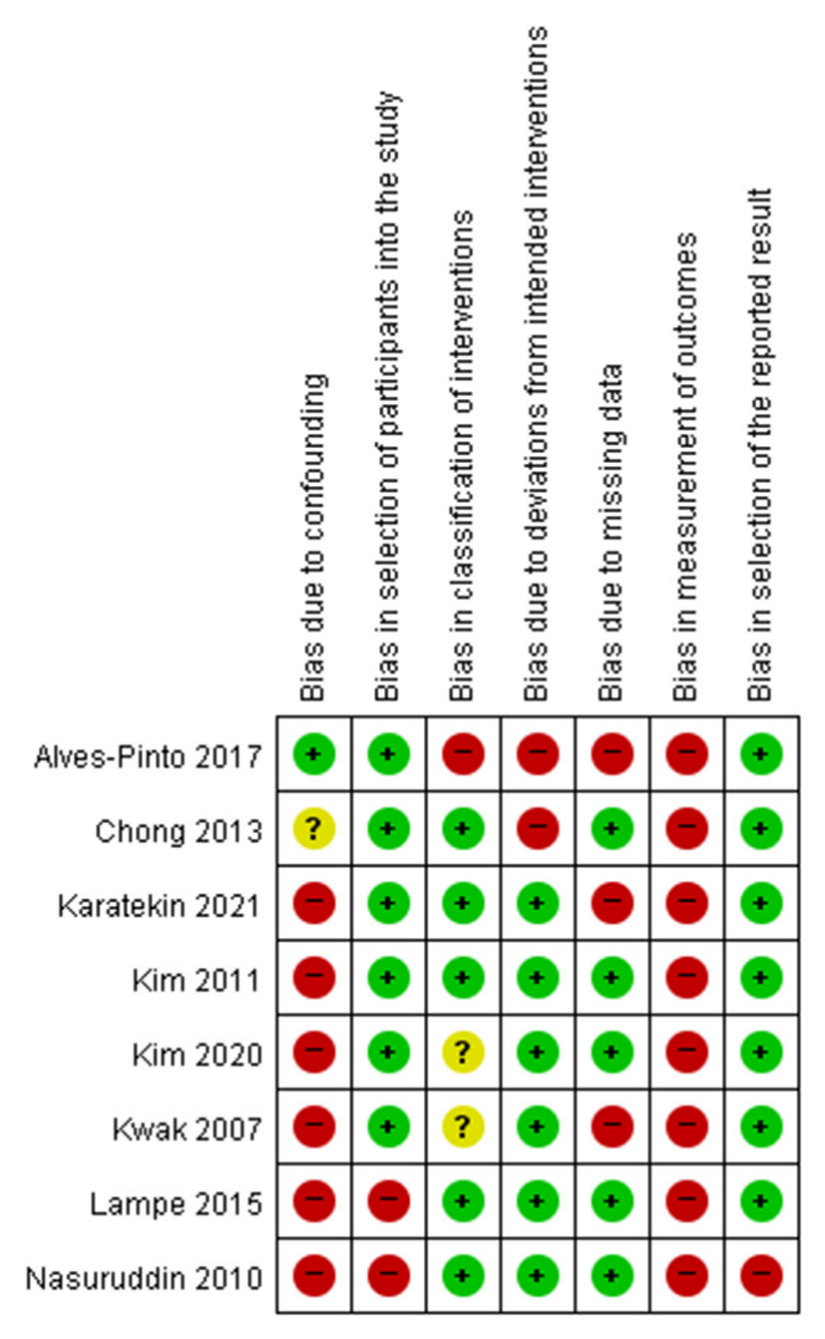
Risk of bias for RCTs.

**Figure 3:**
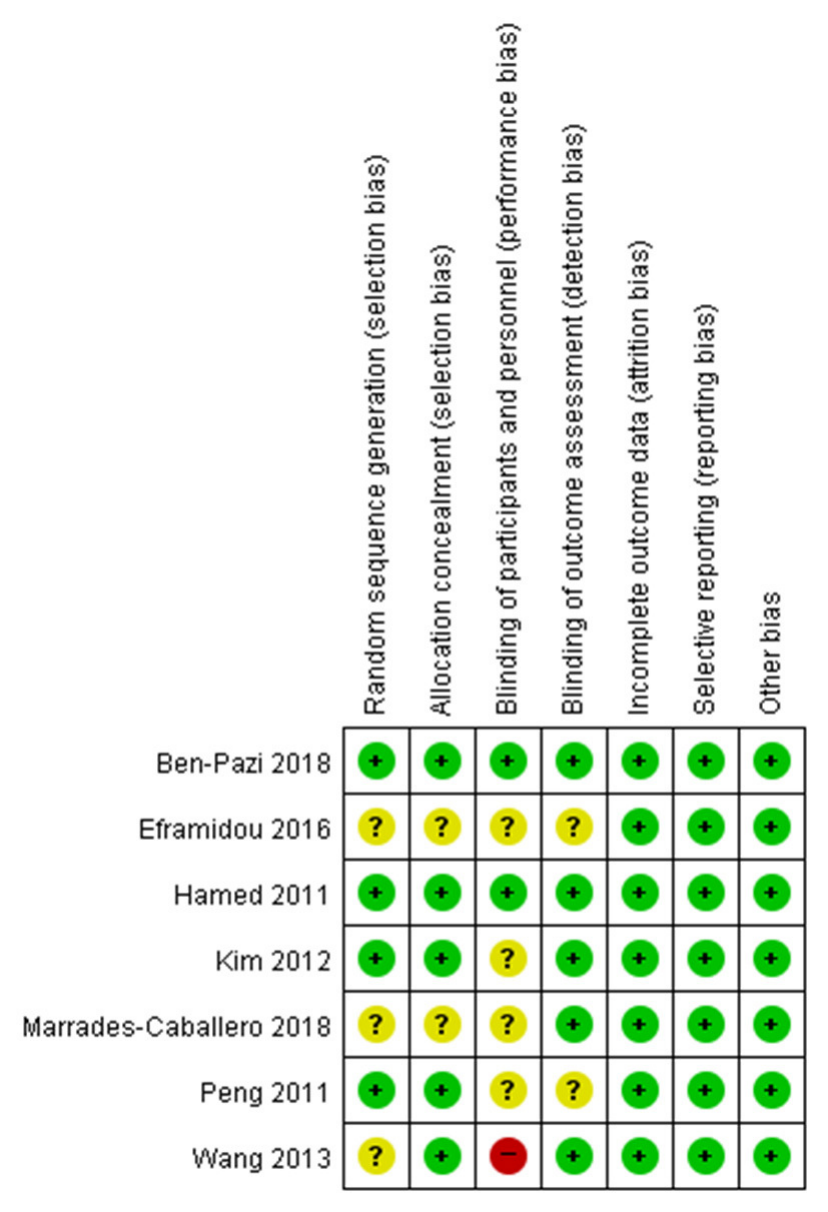
Risk of bias for non-RCTs.

Seven RCTs were assessed using the Cochrane Handbook for Systematic Reviews of Interventions (Figure 2). Notably, regarding the domain for blinding of participants, two studies showed low risk of bias (28, 32) while 4 studies showed unclear risk of bias (12, 30, 31, 37) and one study showed a high risk of bias (38). Eight non-RCTS were assessed using ROBINS-I. Most studies showed high risk of bias in the domains of bias due to confounding and bias in measurement of outcomes (Figure 3).

#### Rhythmic auditory stimulation

The potential use of RAS as a therapeutic strategy for gait training in patients with CP was suggested by the 2007 study of Kwak, which showed that RAS improved gait performance in 18 patients with CP (27). Patients who received gait training with RAS showed a statistically significant difference in stride length, velocity, and symmetry (*P* < 0.05). This study also suggested that other factors, such as physical ability, cognitive functioning, and support of parents, may have influenced on the effectiveness of RAS and outcomes of the training.

In 2011, Kim et al. enrolled 14 adult patients with CP and compared the effect of RAS on gait patterns with 30 healthy controls (29). RAS induced kinematic changes in anterior tilt of pelvis and hip flexion during a gait cycle and improved gait deviation index. Household ambulators, who were limited to walking indoors only short distances either independently or with assistive device, showed that RAS was associated with improvement in gait pathology and temporospatial asymmetry. In the same year, Hamed and Abd-elwahab investigated the effect of music using pedometer (28). Thirty children with CP received pedometer-based or conventional gait training program. A pedometer was fastened on a belt or waistband, while it played seven melodies while walking and jogging and gave motivation and auditory feedback. The tempo synchronized with walking speed and the patient was encouraged to improve the rhythm and speed. The results showed that pedometer-based gait training was effective as all the gait parameters, including velocity, stride length, and cadence, showed statistically significant improvement in patients who received musical cues compared with the controls.

In 2012, Kim et al. enrolled 28 patients with CP and compared the effect of RAS with neurodevelopmental treatment (NDT, 15 vs. 13 patients) (30). Patients in both groups received treatment sessions of 30 min, three times a week for 3 weeks. Fifteen patients in NDT group received gait training following the traditional method, whereas other 13 patients received RAS in a simple rhythm pattern synchronized with the beats of a metronome, based on the individual's cadence. The results showed that functional gait measures, including cadence, stride length, step length, stride time, step time, and walking velocity, significantly improved after gait training with RAS. The anterior tilt of the pelvis and hip flexion during a gait cycle was significantly improved after RAS, in addition to improvement in gait deviation index in patients treated with RAS compared with NDT.

A study on the effect of RAS was also conducted by Efraimidou and Proios in 2016 (31). In this study, 10 male athletes with CP were divided randomly into music group (5 patients) and control group (5 patients). Patients in the music group received 50 min of music and movement program, which included gait and balance with music exercises based on RAS method, twice a week for 8 weeks. During the program, patients were encouraged to sing to improve mood, perform stretching exercises with music tracks of 4/4 music meter and music tempo of 70 beat per minute, walk to the rhythm of 4/4 with the tempo of 90 beat per minute, and repeat relaxation exercises with the tempo of 70 beat per minute. Compared with the control group who received regular training, patients in the music group showed statistically significant changes in gait, balance, and psychological parameters after the program.

A 2018 study of Ben-Pazi et al., compared 9 children with CP who received auditory stimulation embedded in music with 9 children with CP listened to music alone (32). Children who received auditory stimulation attained more goals than children who listened to music alone. Parents reported better care and comfort in children in the intervention group as well as better upper extremity skills. No significant change was observed in gross motor function in both groups.

Most recently, in 2020, Kim et al. investigated the effect of RAS by comparing the use of simple vs. complex chords (33). Thirteen adult patients with CP received gait-training program with RAS, which consisted 30-min sessions, a three times a week for 4 weeks. Six patients in the simple RAS group received basic chords for cueing and seven patients in the complex RAS group received diversified chords. After RAS, spatiotemporal gait parameters including velocity, cadence and stride length increased significantly, but no differences were observed between the groups. Interestingly, patients in the complex RAS group showed significantly greater ankle plantar flexion at push-off than patients in the simple RAS group. This study supported that rhythm is important for gait control, and that the level of complexity of music, which affects perception of music, may have influenced dynamic ankle movement.

#### Therapeutic instrumental music performance

Seven studies used musical instruments in patients with CP to improve their motor and functional skills. Most studies used piano or keyboard as an instrument for TIMP.

In 2010, Nasuruddin enrolled 9 children with CP and applied therapeutic intervention including Gamelan music and theater games (34). This type of music includes percussive instruments such as circular knobbed gongs and bronze plates of varying pitches, which do not require technical skills. Children were instructed to play the Gamelan instruments and drums. After therapy sessions, all children showed significantly higher scores on gross motor function, as measured by improvement in standing. They also showed significant improvement over time on the measures of walking, jumping, and running.

The improvement in hand function was observed in 5 adults with CP in the 2013 study of Chong et al. (35). Keyboard training session was conducted for 30 min, twice a week for a maximum of 9 weeks and hand function was measured using Musical Instrument Digital Interface (MIDI), which is used for the analysis of the velocity and force of the keystrokes. This study suggested that TIMP using keyboard playing may effectively improve manual dexterity and velocity of finger movement. The velocity of key pressing force increased to a significant level, and the second and fifth fingers improved to a greater degree after keyboard training.

In 2015, Lampe et al. included 10 children with CP who had impaired hand motor function to investigate whether learning to play the piano helped to improve their hand motor skills (36). Patients were trained by a professional piano teacher for about 40 min twice a week for 18 months. The uniformity of finger strokes was objectively assessed from the timing of keystrokes using MIDI. Although no significant changes were detected by the Box-and-Block test and grip strength test after training, a significant improvement in the uniformity of keystrokes were seen in patients after piano training with strong motivation and engagement during the training.

A 2017 study of Alves-Pinto et al. investigated the effect of piano training for 16 adolescents and adults with CP (7). This study reported significant effects of training on the ability to perceive the localization of vibrations over fingers, but no significant change was detected on the performance of simple finger tapping sequences on the piano. This study suggested that a longer period of training for more than 4 weeks or 2 h of training per week, may be needed to detect changes in finger movement performance.

In 2018, Marrades-Caballero et al. enrolled 18 patients with severe CP and demonstrated that they could benefit from TIMP program in addition to usual physiotherapy (12). Significant improvement in arm and hand position, activities, and locomotor stages were observed (*P* < 0.05) after handling musical instruments through TIMP, showing the development of manual function. The optimized intervention of TIMP can improve upper limb function in children with severe bilateral CP and this effect lasted 4 months after the treatment.

Most recently, in 2021, Dogruoz Karatekin and Icagasioglu investigated the effect of TIMP in 9 adolescents with piano training sessions, consisting 40 min of training twice a week for 3 months (25). After piano training, significant improvements were observed in Manual Ability Classification System, box block test, nine-hole peg test, and key pressing power of all fingers, especially 4th and 5th fingers. The authors suggested that active music production with TIMP method can bring functional gains by improving grip strength, selective strengths of the fingers, and gross and fine hand motor skills.

#### Patterned sensory enhancement

Two studies used PSE as a therapeutic technique in patients with CP and reported the positive effect of PSE. In 2011, Peng et al. conducted a study involving 23 patients with CP (37). This study focused on improvement in sit-to-stand (STS) exercise, which is a common functional strengthening exercise for lower extremities. Under music condition, individualized PSE music composed based on each patient's STS movement was played during the first five repetitions of STS, and the effect was compared with the control condition. In the PSE condition, improvement in peak knee extensor power (*P* = 0.009), total extensor power (*P* = 0.015), and center-of-mass smoothness (*P* = 0.01) were observed, which showed that individualized PSE music helped to improve the performance of STS.

The effect of PSE music was also investigated by Wang et al. in 2013 (38). Thirty-six children with CP were allocated into PSE group (18 patients) and control group (18 patients), to perform loaded STS exercises three times a week for 6 weeks. PSE music was prescribed individually and all PSE music samples were composed by a music therapist. The results showed that the PSE group improved significantly more than the control group in the Gross Motor Function Measures (GMFM) score after training and the improvement persisted for at least 6 or 12 weeks.

## Discussion

Patients with CP have impairment in the development of movements and postures, difficulties in performing activities (1). Impaired mobility and dependence in activities of daily living have great impact on quality of life and social activities and these are important issues for patient with CP (33). For these patients, effective rehabilitation programs are necessary to promote the use of hands for daily activities and preserve locomotor abilities (36). Rehabilitation programs play an important role through several therapeutic interventions to achieve better motor and functional outcome in patients with CP (39). Previous studies have suggested that the use of NMT in combination with physical therapy improves motor function in patients with CP (1, 40). NMT helps to activate motor and auditory system, facilitating improvement in balance, walking, and mental health conditions (41). Our review included 15 studies that investigated various techniques of NMT, such as RAS, TIMP, and PSE. RAS was effective in improving gait parameters and TIMP was effective in improving gross and fine motor skills, especially hand function and key pressing power. PSE was also helpful to enhance improvement in gross motor capacity.

NMT induces positive effects during rehabilitation through repetition, organization, participation, reward, and motivation. It improves motor control through neurophysiological processes such as priming and entrainment (9, 13, 42). For example, RAS is used to construct a temporal template in the auditory system for movement control, thereby implementing auditory-motor entrainment. Rhythm is a primary agent for synchronization of conscious repetitive movement (33). Rhythm synchronizes movement, modulates muscle activation patterns, and controls movement in space (12). The function of rhythmic entrainment to promote improvement in human sensory and motor systems in rehabilitative training and learning was first established by Thaut et al. (43). It was suggested that entrainment cues, such as auditory rhythmic patterns, can change the timing of movement, improve spatial and force parameters, and optimize motor planning and execution (9).

RAS uses regularly timed external auditory stimulation during gait training (33). External cueing is applied during gait training of patients with neurological disease to help synchronize heel strikes. These repetitions of external timing auditory cues are provided to regulate movement patterns, elicit muscular activation in a step-by-step formation and induce a desirable motor movement to achieve a complete target movement (35). Seven studies that investigated the effect of RAS in patients with CP were identified in our review (27–31, 33). These studies showed that gait training with RAS is effective to promote faster walking, better pelvic and hip movements, and gait control in patients with CP.

Gait training with RAS involves musical properties, and uses metronomes or original recorded music. RAS is associated with improvement in spatiotemporal gait parameters such as walking speed and length of stride, and kinematic parameters such as joint movements during a gait cycle (33). External auditory cues activates subcortical neuronal loops, which control balance and motor coordination (44, 45). RAS aims to facilitate auditory-motor synchronization and regulate the reticulospinal pathway in the brain (30). It is postulated that repetitive rhythmic sound patterns induce the excitability of spinal motor neurons through the reticulospinal pathway, entraining the coordination of axial and proximal movement to a given motor command (46).

TIMP addresses motor rehabilitation by using musical instruments to train motor functions and facilitate functional movement patterns (7, 12). TIMP uses musical instruments to induce and strength appropriate functional motor patterns. The goals of TIMP are to improve coordination, range of motion, manipulation, and finger dexterity including palmar and pincer grasp (31). After search, seven studies were found which investigated the effect of TIMP in patients with CP (7, 12, 25, 32, 34–36). These studies showed the potential of playing musical instruments as a rehabilitation strategy to enhance motor skills.

Playing an instrument involves repetition of fine finger movements, finger and hand coordination, and auditory feedback. After receiving an immediate auditory feedback, the player tries to process his performance and tries to correct it. By doing so, he trains fine motor skills and integrates audio-visual information with motor control and coordinates finger and hand movements (36). TIMP aims to facilitate movement based on non-musical rehabilitative purposes, not on production of great music (35). Selection of appropriate instruments and application of playing techniques are important to achieve these goals. Playing an instrument such as piano or keyboard helps to improve manual skills through continuous fine motor skill training. Playing a musical instrument requires multiple skills. It engages integration of auditory, visual, and somatosensory information and motor coordination. Playing music also incorporates the entertaining and joyful component from making audible music, in addition to the drive for mastering a music instrument (7). The emotional and entertaining effects of music provides motivation to patients, leading to active participation in rehabilitation treatment, which may increase the efficacy of therapy (36).

Two studies on the effect of PSE in patients with CP were included in this review. The goal of PSE is to translate movement patterns into sound patterns in order to give the spatial, temporal, and force cueing. PSE uses rhythm, pitch, dynamics to regulate movement patterns to facilitate movement execution (38). Pitch variation, such as ascending and descending melodic lines, can guide direction of a movement. Rhythmic pattern, tempo, and meter can give guidance to the timing and speed of a movement. Volume and harmony can induce further strength of muscle activation. Theses musical elements are used commonly in PSE to create a sound pattern to cue appropriate execution of movement (38).

### Limitations

The current review investigated the effect of NMT in patients with CP, and this review has some limitations. Some studies compared the effect of NMT with controls (no-CP controls or no-NMT controls) (7, 12, 25, 27–29, 31, 32, 38). In contrast, other studies were observational studies without a control group for comparison (30, 34–37). In addition, one study compared specific methods of RAS (simplex vs. complex chords) (33) and another study compared RAS with traditional method of rehabilitation (NDT) (30). This review is also limited due to the small sample size of included studies. Relatively small number of patients (about 10–30 patients) were included to investigate the effect of NMT in these studies, and a small sample of patients may restrict the generalization of the effect of NMT. In addition, it is possible that many confounding factors, such as age, type of CP, duration of the treatments, or evaluation tools, could have affected the clinical outcomes of CP, and the studies included in this review did not consider all these possible confounding factors. Designs were also different across studies. Studies were selected regardless of age and the age groups of the patients varied among studies, and some studies did not provide full description of the methods of their intervention, including the frequency and duration of the intervention. The number of studies on various types of NMT were small and sessions of NMT varied among studies, thus, we could not conduct a meta-analysis to summarize the overall effect of NMT. Due to heterogeneous designs of these studies, the overall body of evidence of NMT in CP patients seems to be either low or insufficient. A larger sample is needed to investigate how age, subtypes of CP, spasticity, muscle power, and functional level can affect the effect of NMT. Also, further studies on effective number of sessions, including duration, frequency, and intensity of therapy are needed in the future.

## Conclusion

Various techniques of NMT brings beneficial effects for gross and fine motor improvements in patients with CP. NMT techniques, such as RAS, TIMP, and PSE, may be a potential alternative rehabilitation strategy to enhance gross and fine motor skills for patients with CP.

## Data availability statement

The original contributions presented in the study are included in the article/Supplementary material, further inquiries can be directed to the corresponding author/s.

## Author contributions

SY and MC: conceptualization and methodology. JS and SK: writing-original draft. SY: writing-review. MC: editing and supervision. All authors contributed to the article and approved the submitted version.

## Funding

This work was supported by the Ewha Womans University Research Grant of 1-2021-1794-001-1 and the National Research Foundation of Korea Grant funded by the Korean Government (Grant No: NRF-2019M3E5D1A02069399).

## Conflict of interest

The authors declare that the research was conducted in the absence of any commercial or financial relationships that could be construed as a potential conflict of interest.

## Publisher's note

All claims expressed in this article are solely those of the authors and do not necessarily represent those of their affiliated organizations, or those of the publisher, the editors and the reviewers. Any product that may be evaluated in this article, or claim that may be made by its manufacturer, is not guaranteed or endorsed by the publisher.

## Abbreviations

CP: cerebral palsy
GMFM: Gross Motor Function Measure
NDT: neurodevelopmental treatment
PSE: patterned sensory enhancement
RAS: rhythmic auditory stimulation
TIMP: therapeutic instrumental music performance
STS: sit-to-stand exercise.
