# Supplementary material for: The effect of neurologic music therapy in patients with cerebral palsy: A systematic narrative review
Source: Front Neurol. 2022 Sep 13;13:852277. doi: 10.3389/fneur.2022.852277 (PMC9514322; doi:10.3389/fneur.2022.852277)
Supplement: Supplementary file 1 [file Data_Sheet_1.docx]

**Supplementary Material**

**Search strategy**

A search strategy was developed to identify studies evaluating the effect of neurologic music therapy on cerebral palsy.

The search keywords were as follows:

'music*,’ ‘music therapy,’ ‘neurologic music therapy,’

‘cerebral palsy,’ ‘cerebral pals*,’ ‘CP’

The search keywords were devised using a combination of subject indexing terms in the titles and abstracts. For the index related to music therapy was referenced [*Schmid W, Rosland JH, von Hofacker S, Hunskår I, Bruvik F. Patient's and health care provider's perspectives on music therapy in palliative care - an integrative review. BMC Palliat Care. 2018 Feb 20;17(1):32.*] and [*Cowen B. Neurologic music therapy techniques: a systematic review of current research. 2014.*]. For the index related to cerebral palsy was referenced [*Johansen T, Strøm V, Simic J, Rike PO. Effectiveness of training with motion-controlled commercial video games on hand and arm function in young people with cerebral palsy: A systematic review and meta-analysis. J Rehabil Med. 2019 Dec 3;52(1):jrm00012.*]

**Search strategy for:** Patient's and health care provider's perspectives on music therapy in palliative care - an integrative review.

| Schmid W, Rosland JH, von Hofacker S, Hunskår I, Bruvik F. Patient's and health care provider's perspectives on music therapy in palliative care - an integrative review. BMC Palliat Care. 2018 Feb 20;17(1):32.  **Table 1.** Search strategy  1. palliative care/ or terminal care/ or hospice care/ or terminally ill/  2. palliative care.mp. or exp. Palliative Care/  3. terminal care.mp. or Terminal Care/  4. exp. Hospice Care/  5. Hospice Care.mp. or Hospice Care/  6. exp. Terminally Ill/  7. terminally ill.mp. or Terminally Ill/  8. hospice*.tw.  9. (palliat* or (terminal* adj6 ill*) or (terminal* adj3 care) or (end adj3 life)).tw.  10. ((care adj5 dying) or (caring adj5 dying) or (support$ adj5 dying) or (dying adj5 patient$)).tw.  11. 1 or 2 or 3 or 4 or 5 or 6 or 7 or 8 or 9 or 10  12. music therapy.mp. or exp. Music Therapy/  13. music*.mp.  14. melody.mp.  15. (music$ or melod$).tw.  16. (sing or sings or singer$ or singing or song$).tw.  17. 12 or 13 or 14 or 15 or 16  18. 11 and 17  19. protocol*.tw.  20. 18 not 19  21. limit 18 to yr. = “1978 -Current” |
| --- |

**Search strategy for:** Neurologic music therapy techniques: a systematic review of current research.

| Cowen B. Neurologic music therapy techniques: a systematic review of current research. 2014.  **CHAPTER II: Methods**  The search terms include: music therapy, neurologic music therapy, NMT, cognitive rehabilitation, speech and language rehabilitation, musical mnemonics training, gait rehabilitation, physical deficits, visual neglect, mood and memory training, rhythmic auditory entrainment, instruments, therapeutic instrument playing, gait training, sonification, melodic intonation therapy, music and speech, singing, mood and memory, rhythmic cueing, vocal intonation, oral motor and respiratory, speech motor control, dysarthria, stroke, dementia, arousal orientation, attention maintenance, inattention, sensory integration, integrating movement to music, and executive function and music. |
| --- |

**Search strategy for:** Effectiveness of training with motion-controlled commercial video games on hand and arm function in young people with cerebral palsy: A systematic review and meta-analysis.

| Johansen T, Strøm V, Simic J, Rike PO. Effectiveness of training with motion-controlled commercial video games on hand and arm function in young people with cerebral palsy: A systematic review and meta-analysis. J Rehabil Med. 2019 Dec 3;52(1):jrm00012.  **Appendix SII.** Medline search strategy  1. Cerebral Palsy/  2. (cerebral pals* or CP or cerebral paresis).tw,kf.  3. 1 or 2  4. Video Games/  5. user-computer interface/  6. (exergame* or game* or gaming or videogam* or nintendo or playstation or xbox or virtual reality or computer play or neurogame* or wii or kinect).tw,kf.  7. 4 or 5 or 6  8. 3 and 7  9. exp Upper Extremity/  10. (upper limb* or arm* or hand* or finger* or wrist* or upper extremit* or forearm* or shoulder* or elbow*).tw,kf.  11. 9 or 10  12. 8 and 11 |
| --- |

**Selection of eligible studies**

We searched trial registers using the 'music*,’ ‘music therapy,’ ‘neurologic music therapy,’ ‘cerebral palsy,’ ‘cerebral pals*,’ ‘CP’ index, and databases including PubMed, Embase, Scopus, Cochrane library, Web of science, and Ovid MEDLINE, for studies published up to May 24, 2022. The results of the database searches were entered into an EndNote X9 library. Duplicates were deleted using the deduplication function in EndNote X9. Two reviewers preferentially removed irrelevant records for selection based on the titles and abstracts. Thereafter, the reviewers checked the full texts to finally select the papers that meet the selection criteria.

Each database was searched under the following conditions:

1. PubMed (674)
   - Subject of study: human

- Language: English

1. Embase (218)
   - Subject of study: human

- Language: English

1. Scopus (191)

- Language: English

1. Cochrane library (51)
   - Study types: trials
2. Web of science (845)
   - Language: English
3. Ovid MEDLINE (2138)
   - Subject of study: human

- Language: English

- Publication types: clinical study; clinical trial, All; clinical trial; controlled clinical trial; observation study

**Eligibility criteria**

| **Population** | We included studies in patients with cerebral palsy treated with music therapy. |
| --- | --- |
| **Intervention** | A paper evaluating the results of neurologic music therapy was included. |
| **Comparison** | No specific comparison target has been established. |
| **Outcome** | Studies were considered eligible for inclusion in this review if they reported on the improvement of gross motor, fine motor, and function. |
| **Study design** | Studies published as reviews, case reports, commentaries, letters, and animal studies were excluded. |
| **Limitation** | Publications in a language other than English were excluded. |
